# 2015–2016 Vaccine Effectiveness of Live Attenuated and Inactivated Influenza Vaccines in Children in the United States

**DOI:** 10.1093/cid/cix869

**Published:** 2017-10-04

**Authors:** Katherine A Poehling, Herve Caspard, Timothy R Peters, Edward A Belongia, Blaise Congeni, Manjusha Gaglani, Marie R Griffin, Stephanie A Irving, Poornima K Kavathekar, Huong Q McLean, Allison L Naleway, Kathleen Ryan, H Keipp Talbot, Christopher S Ambrose

**Affiliations:** 1 Wake Forest School of Medicine, Winston-Salem, North Carolina; 2 MedImmune, Gaithersburg, Maryland; 3 Marshfield Clinic Research Institute, Marshfield, Wisconsin; 4 Akron Children’s Hospital, Ohio; 5 Baylor Scott & White Health, Texas A&M Health Science Center College of Medicine, Temple; 6 Vanderbilt University Medical Center, Nashville, Tennessee; 7 Kaiser Permanente Center for Health Research, Portland, Oregon; 8 HealthPartners Como Clinic, St Paul, Minnesota; 9 University of Florida, Gainesville

**Keywords:** children, influenza, influenza vaccine, vaccine effectiveness

## Abstract

**Background:**

In the 2015–2016 season, quadrivalent live attenuated influenza vaccine (LAIV) and both trivalent and quadrivalent inactivated influenza vaccine (IIV) were available in the United States.

**Methods:**

This study, conducted according to a test-negative case-control design, enrolled children aged 2–17 years presenting to outpatient settings with fever and respiratory symptoms for <5 days at 8 sites across the United States between 30 November 2015 and 15 April 2016. A nasal swab was obtained for reverse-transcriptase polymerase chain reaction (RT-PCR) testing for influenza, and influenza vaccination was verified in the medical record or vaccine registry. Influenza vaccine effectiveness (VE) was estimated using a logistic regression model.

**Results:**

Of 1012 children retained for analysis, most children (59%) were unvaccinated, 10% received LAIV, and 31% received IIV. Influenza A (predominantly antigenically similar to the A/California/7/2009 strain) was detected in 14% and influenza B (predominantly a B/Victoria lineage) in 10%. For all influenza, VE was 46% (95% confidence interval [CI], 7%–69%) for LAIV and 65% (48%–76%) for IIV. VE against influenza A(H1N1)pdm09 was 50% (95% CI, −2% to 75%) for LAIV and 71% (51%–82%) for IIV. The odds ratio for vaccine failure with RT-PCR–confirmed A(H1N1)pdm09 was 1.71 (95% CI, 0.78–3.73) in LAIV versus IIV recipients.

**Conclusions:**

LAIV and IIV demonstrated effectiveness against any influenza among children aged 2–17 years in 2015–2016. When compared to all unvaccinated children, VE against influenza A(H1N1)pdm09 was significant for IIV but not LAIV.

**Clinical Trials Registration:**

NCT01997450

## Teaser

This test-negative case-control study showed live attenuated influenza vaccine and inactivated influenza vaccine were effective against any influenza in 2015–2016.

**(See the Editorial Commentary by Ortiz on pages 673–5.)**

Influenza is a common cause of respiratory illness, resulting in billions of dollars in both direct and indirect medical costs across the United States [1]. Influenza-associated outpatient visits occur in all age groups, with the highest annual rates among children aged 2–17 years [2]. In the community, influenza is seen earlier among school-aged children and young adults than in other age groups [3, 4].

Annual influenza vaccination has been recommended for children aged 6 months to 18 years in the United States since 2008 [5] and has been demonstrated to decrease the risk of inpatient and outpatient influenza illnesses among vaccinated persons [6]. Indirect protection of children has also been assessed through school-based vaccination programs and among the Hutterite community [7, 8].

This study was initiated in 2013–2014 to estimate the vaccine effectiveness (VE) of quadrivalent live attenuated influenza vaccine (LAIV) and inactivated influenza vaccine (IIV) among children over 4 consecutive seasons. This report focuses on the 2015–2016 season and follows VE reports from the 2 previous seasons [9, 10].

In June 2014, LAIV was preferentially recommended by the Centers for Disease Control and Prevention (CDC) Advisory Committee on Immunization Practices (ACIP) for healthy children aged 2–8 years because clinical studies demonstrated superior VE with trivalent LAIV than with trivalent IIV [11–13]. However, this preferential recommendation was subsequently removed because of the low effectiveness of quadrivalent LAIV in 2013–2014 against A(H1N1)pdm09 viruses [14, 15].

## METHODS

This postmarketing, observational, multiseason study (ClinicalTrials.gov identifier: NCT01997450) was conducted during the 2015–2016 influenza season following the same methods used in the 2013–2014 and 2014–2015 seasons [9, 10]. Four enrolling sites in Florida, Minnesota, Ohio, and Oregon were added for the 2015–2016 influenza season. Community-dwelling children aged 2–17 years seeking outpatient medical care for febrile acute respiratory illness were recruited. Children were eligible for enrollment if they presented with an acute respiratory illness with fever (oral temperature ≥37.8°C at study visit, parental report of fever with or without documented measurement of temperature, or use of antipyretic medication before study visit), with symptom duration of <5 days. Children were excluded from the analyses if they were enrolled before or after influenza circulation at the site, treated with antiviral medication <14 days before enrollment, vaccinated <14 days before symptom onset, received an unknown vaccine type, or reported receipt of vaccine that was not confirmed in the medical record or immunization registry. 

Enrollment began at each site either before the onset of influenza activity or when there was evidence of increasing influenza incidence based on clinical laboratory testing. Enrollment ended in April or when a substantial decline in influenza-positive cases was observed. At each site, influenza-negative controls who were enrolled before the first influenza-positive case or after the last influenza-positive case were excluded from the analysis.

Study recruitment occurred at 8 sites across the United States: Baylor Scott & White Health (Temple, Texas), Marshfield Clinic Research Institute (Marshfield, Wisconsin), Vanderbilt University Medical Center (Nashville, Tennessee), Wake Forest School of Medicine (Winston-Salem, North Carolina), Akron Children’s Hospital (Akron, Ohio), HealthPartners Como Clinic (St Paul, Minnesota), Kaiser Permanente Center for Health Research (Portland, Oregon), and the University of Florida Primary Care Clinics (Gainesville). The study also included sites in the United Kingdom in this season. However, enrollment at those sites (50 children with complete vaccination data) was considered insufficient to yield meaningful VE estimates; UK data were not combined with data from US sites because children were recruited in a different setting.

This US study was reviewed and approved by the institutional review boards at all participating sites. Written informed consent was obtained from the child’s parent/guardian and assent was obtained from the child, when age appropriate, before enrollment. Parents/guardians completed a standardized enrollment questionnaire to ascertain patient demographics and medical history. Data on the number of healthcare visits within the past year and health insurance status were electronically extracted from the medical record.

A nasal swab specimen was collected at the enrollment visit and tested for influenza, respiratory syncytial virus, human metapneumovirus, parainfluenza viruses 1–3, rhinovirus, and adenoviruses B, C, and E at a central laboratory (LabCorp) using a multiplex reverse transcriptase–polymerase chain reaction (RT-PCR) assay (GenMark eSensor; GenMark Diagnostics). Influenza hemagglutinin was sequenced from clinical influenza isolates (cell culture isolates) and subjected to phylogenetic analysis. Influenza strains were then grouped by genetic clade by MedImmune staff who were blinded to vaccine status. It was determined that, during the 2015–2016 season, nearly all influenza A(H1N1)pdm09 viruses in the United States were from genetic subgroups (clades) 6B.1 and 6B.2, with the majority in the 6B.1 clade [16]. Antigenic characterization by the CDC has determined that viruses in the 6B.1 clade were antigenically similar to influenza A/California/7/2009 vaccine reference strain [16, 17]. Therefore, we used genetic clade as a surrogate for antigenic similarity in this analysis.

Influenza vaccination dates and vaccine type from the 2014–2015 and 2015–2016 seasons were obtained from medical record reviews and/or immunization registries. Children were considered vaccinated if they received ≥1 dose of 2015–2016 vaccine ≥14 days before illness onset. Vaccinated children were further classified by type of vaccine received (quadrivalent LAIV, trivalent IIV, or quadrivalent IIV). The 2015–2016 season northern hemisphere trivalent IIV included A/California/7/2009 (H1N1)–like virus, a new A/Switzerland/9715293/2013 (H3N2)–like virus, and a new B/Phuket/3073/2013-like virus (Yamagata lineage). Quadrivalent IIV was similar to trivalent IIV and also included B/Brisbane/60/2008-like virus (Victoria lineage) [18]. LAIV was similar to quadrivalent IIV except that it contained A/Bolivia/559/2013, a more thermostable A/California/7/2009 (H1N1)–like virus than the LAIV strain used in 2013–2014 and 2014–2015.

This study was conducted according to a test-negative, case-control design [19, 20]. Case patients were defined as children with medically attended febrile acute respiratory illness confirmed as influenza by RT-PCR, and controls were children who tested negative for influenza virus. Logistic regression models were used to estimate odds ratios (ORs), comparing unvaccinated children with those vaccinated with quadrivalent LAIV and either quadrivalent or trivalent IIV. VE was calculated as 100% × [1 − OR]. The same model was used to assess effectiveness against any influenza, influenza A(H1N1)pdm09, influenza B, and influenza B/Victoria. 

The odds of vaccine failure (ie, RT-PCR–confirmed influenza) were compared for LAIV recipients versus all IIV recipients. Age group (2–4, 5–8, or 9–17 years), enrollment site, and calendar time (modeled as a series of dichotomous variables representing 4-week intervals) were included in the adjusted model a priori. Sex, number of outpatient visits in the past year, and health insurance status were also included in the adjusted model, because their inclusion changed ≥1 effectiveness estimate for LAIV or IIV by an absolute difference of 3% or more—the predetermined threshold for inclusion in the model. Sensitivity analyses were conducted after exclusion of: (1) partially vaccinated children, defined as those aged <9 years who had not been vaccinated since 2010–2011 and who received only 1 dose in 2015–2016 [18]; (2) controls who tested negative for any respiratory virus; and (3) children with high-risk health conditions. A post hoc sensitivity analysis was performed excluding sites that enrolled <30 children (Florida, Minnesota, Ohio, and Oregon).

A sample of 1300 children was expected to be enrolled each season for the 4-year study. The total sample size of 5200 over 4 years was calculated to provide 99% power to show LAIV effectiveness versus no vaccine and 83% power to show relative LAIV effectiveness versus IIV (assuming 75% LAIV effectiveness, a 23% positivity rate, and proportions of LAIV recipients, IIV recipients, and unvaccinated children of 30%, 30%, and 40%, respectively). However, power was not prespecified for each individual season owing to the unpredictable epidemiology of influenza, including incidence, subtype distribution, and antigenic characteristics of circulating viruses. Statistical analyses were conducted using SAS statistical software (version 9.3; SAS Institute).

## RESULTS

A total of 1254 children were enrolled at 8 sites. Three sites began enrollment in 2015: Wisconsin (starting 30 November), Texas, and Tennessee (both starting 29 December). Five sites began enrollment in 2016: North Carolina (starting 13 January), Oregon (starting 20 January), Minnesota (starting 1 February), Ohio (starting 22 February), and Florida (starting 10 March). All sites closed enrollment on 15 April 2016. Overall, 1012 children (81%) were retained in the analysis. Reasons for exclusion included not meeting eligibility criteria (n = 7), refusal of the nasal swab specimen (n = 9), enrollment before the first or after the last documented influenza detection at that site (n = 215), vaccinated <14 days before symptom onset (n = 7), missing vaccine date or type (n = 3), and lack of a signed Health Insurance Portability and Accountability Act consent form (n = 1).

Among the 1012 children included, 59% were unvaccinated, 10% received LAIV, 10% received trivalent IIV, 20% received quadrivalent IIV, and 1% received IIV of unknown valence (Table 1). There were significant differences between unvaccinated children, LAIV recipients, and IIV recipients by age group, race/ethnicity, enrollment site, high-risk conditions, health insurance, and frequency of outpatient visits. At enrollment, two-thirds of children had symptoms for 2–5 days, with nausea (87%), cough (84%), and congestion (81%) the most prevalent symptoms. Headache was the only symptom that varied by vaccine status (unvaccinated, 59%; LAIV, 47%; IIV, 51%; *P* = .02); a shorter duration since symptom onset was observed among IIV recipients (*P* = .05).

**Table 1.** Study Population Characteristics By Vaccination Status

Characteristic	Children by 2015–2016 Influenza Vaccine Status, No. (%)			*P* Value^a^
	Unvaccinated (n = 594)	LAIV (n = 101)	IIV (n = 317)	
RT-PCR influenza status				
Influenza A positive	17 (103)	13 (13)	8 (25)	< .001
Influenza B positive	12 (70)	9 (9)	6 (19)	
Influenza negative	71 (421)	78 (79)	86 (273)	
Children testing positive to noninfluenza viruses among the test-negative controls	43 (180/421)	44 (35/79)	51 (139/273)	.10
Age range				
2–4 y	28 (166)	33 (33)	36 (114)	< .01
5–8 y	36 (215)	47 (48)	36 (113)	
9–17 y	36 (213)	20 (20)	28 (90)	
Female sex	46 (273)	54 (55)	44 (139)	.17
Race/ethnicity				
Non-Hispanic white	60 (356)	61 (62)	65 (207)	.05
Non-Hispanic black	16 (98)	10 (10)	16 (51)	
Hispanic white	19 (111)	20 (20)	12 (38)	
Other	5 (29)	9 (9)	7 (21)	
Enrollment site				
Florida	1 (4)	2 (2)	<1 (1)	< .001
Minnesota	2 (13)	2 (2)	1 (3)	
North Carolina	17 (99)	2 (2)	8 (25)	
Ohio	4 (24)	9 (9)	2 (5)	
Oregon	2 (10)	4 (4)	1 (9)	
Tennessee	11 (63)	35 (35)	28 (87)	
Texas	38 (224)	32 (32)	28 (88)	
Wisconsin	26 (157)	15 (15)	31 (99)	
Enrollment period				
3–30 January	18 (105)	12 (12)	15 (47)	.25
31 January to 27 February	36 (216)	35 (35)	31 (99)	
28 February to 26 March	36 (213)	40 (40)	40 (128)	
27 March to 15 April	10 (60)	14 (14)	14 (43)	
Presence of high-risk conditions for influenza	16 (93)	6 (6)	26 (81)	< .001
Privately insured	45 (265)	56 (57)	53 (168)	.01
Fully vaccinated in 2015–2016	…	96 (97)	95 (301)	…
No. of outpatient visits in past 6 mo				
≤1	57 (337)	41 (41)	42 (132)	< .001
≥2	43 (257)	59 (60)	58 (185)	
Reported symptoms				
Fever	100 (594)	100 (101)	100 (317)	>.99
Cough	85 (507)	81 (82)	83 (264)	.48
Sore throat	74 (439)	74 (75)	62 (198)	.001
Runny or stuffy nose	81 (481)	74 (75)	83 (262)	.17
Headaches	59 (350)	47 (47)	51 (163)	.02
Body aches	45 (266)	38 (38)	39 (124)	.16
Irritability	59 (349)	57 (58)	56 (178)	.75
Fatigue/run down	88 (520)	81 (82)	89 (282)	.12
Nausea	35 (208)	37 (37)	38 (120)	.69
Vomiting	28 (169)	19 (19)	24 (77)	.08
Diarrhea	15 (87)	7 (7)	13 (41)	.10
Time since symptom onset <2 d	31 (183)	35 (35)	39 (123)	< .05

Abbreviations: IIV, inactivated influenza vaccine; LAIV, live attenuated influenza vaccine; RT-PCR, reverse-transcriptase polymerase chain reaction.

a *P* value from tests comparing distributions between unvaccinated children, LAIV recipients, and IIV recipients.

**Figure 1.**
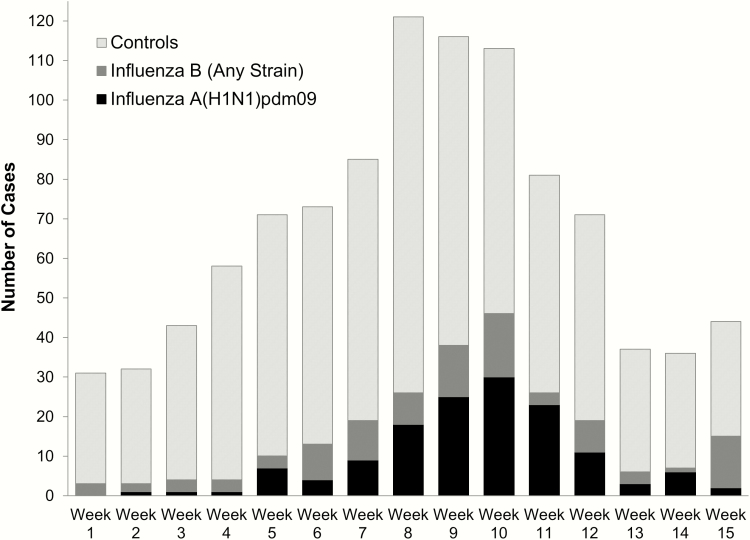
Distribution of influenza cases and controls by week of enrollment.

Influenza A and B were detected in 14% and 10% of children, respectively. Most influenza A cases (99%) were A(H1N1)pdm09 strains. All sequenced influenza A(H1N1)pdm09 viruses belonged to clade 6B.1, consistent with US surveillance data [16], and were antigenically similar to the vaccine strain. Sixty-nine percent of influenza B viruses were Victoria lineage, 21% were Yamagata lineage, and 10% had missing lineage. Most children (72%) were enrolled between calendar weeks 5 through 12 (31 January to 26 March 2016) when the majority of influenza A and B cases were detected overall (Figure 1) and at each site (not shown).

**Figure 2.**
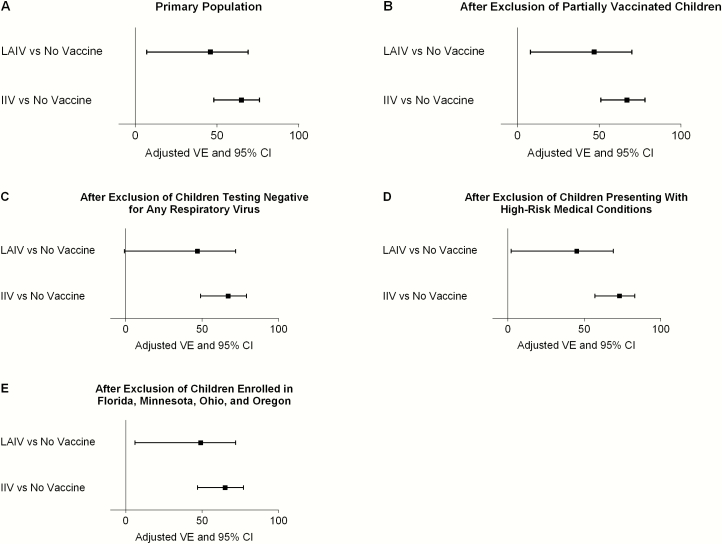
Box-and-whisker plots of live attenuated influenza vaccine (LAIV) and inactivated influenza vaccine (IIV) vaccine effectiveness (VE) against any influenza strain in the primary population (*A*) and after exclusion of partially vaccinated children (*B*), children testing negative for any respiratory virus (*C*), children presenting with high-risk medical conditions (*D*), or children enrolled at sites that enrolled <30 children (Florida, Minnesota, Ohio, and Oregon) (*E*). Abbreviation: CI, confidence interval.

Compared with no vaccination, VE against any influenza was 46% (95% confidence interval [CI], 7%–69%) for LAIV and 65% (48%–76%) for IIV (Table 2 and Figure 2A). VE against influenza A(H1N1)pdm09 strains was 50% (95% CI, −2% to 75%) for LAIV and 71% (51%–82%) for IIV. VE against any influenza B strains was 47% (95% CI, −18% to 76%) for LAIV and 56% (21%–75%) for IIV. More specifically, LAIV and IIV VE against B/Victoria lineage strains were 69% (95% CI, −7% to 91%) and 64% (20%–84%), respectively. The odds of any influenza and influenza A(H1N1)pdm09 among LAIV recipients were higher than in IIV recipients (OR, 1.54 [95% CI, .85–2.79] and 1.71 [.78–3.73], respectively), but the ORs were not significantly different from 1.00. VE estimates by vaccine type (LAIV, trivalent IIV, and quadrivalent IIV) are presented in Supplementary Table S1. 

**Table 2.** 2015–2016 Adjusted VE Estimates Overall and by Predominant Strains by Vaccine Type^a^

Influenza Strain	Unvaccinated Children (n = 594)	LAIV Recipients (n = 101)	IIV^b^ Recipients (n = 317)	VE %, (95% CI)		LAIV4 vs IIV, OR (95% CI)
				LAIV4 vs No Vaccine	IIV vs No Vaccine	
Any influenza (cases)	173	22	44	46 (7–69)	65 (48–76)	1.54 (.85–2.79)
A(H1N1)pdm09	102	12	25	50 (–2 to 75)	71 (51–82)	1.71 (.78–3.73)
Any B strain	70	9	19	47 (–18 to 76)	56 (21–75)	1.20 (.51–2.86)
B/Victoria	48	3	9	69 (–7 to 91)	64 (20–84)	0.85 (.72–3.34)

Abbreviations: CI, confidence interval; IIV, inactivated influenza vaccine; LAIV, live attenuated influenza vaccine; OR, odds ratio; VE, vaccine effectiveness.

a Case patients are influenza-positive children; controls are influenza-negative children.

b IIV includes both trivalent and quadrivalent IIV.

Results for any influenza were similar when partially vaccinated children were excluded and when controls were limited to those with another virus detected (Figure 2B and 2C). Results against any influenza were also similar when case patients and controls were limited to those without high-risk conditions (Figure 2D), but LAIV VE for influenza A(H1N1)pdm09 reached statistical significance at 54% (95% CI, 1%–78%). The post hoc sensitivity analysis found similar results when the sites that enrolled <30 children (Florida, Minnesota, Ohio, and Oregon) were excluded (Figure 2E).

## DISCUSSION

Both LAIV and IIV were protective against any influenza strain in 2015–2016 and against influenza A(H1N1)pdm09 strains in children without a high-risk condition. However, only IIV provided significant protection against influenza A(H1N1)pdm09 strains in the total study population. When LAIV and IIV recipients were directly compared, the odds of influenza A(H1N1)pdm09 infection increased among LAIV recipients. This difference was not statistically significant; however, the direction and magnitude of the association were similar to what was reported by the CDC’s US Influenza Vaccine Effectiveness Network (US Flu VE Network) during the same season [17].

Estimates of VE are important to the ACIP because they help inform the annual recommendations for influenza vaccination in children. The ACIP preferential recommendation for LAIV among children aged 2–8 years in 2014 was based on randomized controlled trials conducted before 2009, in which trivalent LAIV had demonstrated superior efficacy against influenza among children versus trivalent IIV [11–13]. During the 2009 pandemic, monovalent LAIV influenza A(H1N1)pdm09 vaccine was also reported to be effective against medically attended influenza [21, 22]. However, several studies since 2009 have not found LAIV to be effective, including a 2010–2011 study of trivalent LAIV in the US military [23] and 2 studies of quadrivalent LAIV in American children in 2013–2014 [9, 14]. Given these results, the influenza A(H1N1)pdm09 strain was replaced in the LAIV formulation for the 2015–2016 season [24].

We identified 6 additional studies that examined LAIV VE against laboratory-confirmed influenza in children presenting with medically attended acute respiratory illness in the 2015–2016 season [17, 25–29]. One study was conducted among 2-year-old children in Finland according to a cohort design. Five other studies, including the US Flu VE Network study sponsored by the CDC, were conducted according to an observational test-negative case-control design with relatively few differences in the protocol specifications. The study conducted by the US Flu VE Network found that LAIV was not significantly effective against any strain (VE, 5%; 95% CI, −48% to 39%) and against influenza A(H1N1)pdm09 strains (−18%; <−50% to 34%). These findings led the ACIP to make the interim recommendation that LAIV should not be used in the 2016–2017 season [16, 30]. 

Four of the 5 other test-negative case-control studies found that LAIV was significantly effective against any influenza strains, with point estimates ranging from 46% to 74% (VE estimate against any influenza was missing in the study conducted in Germany [27]). Notably, point estimates of VE against influenza A(H1N1)pdm09 strains were consistently lower with LAIV than with IIV. These findings demonstrate the importance of estimating VE not only against all influenza strains but also against the most frequent circulating strains, because influenza vaccine strain circulation varies regionally and changes throughout the season.

Differences between the VE point estimates across the CDC’s US Flu VE Network study and our study were large and need to be interpreted with caution, given the limited sample sizes and the relatively large 95% CIs. Both studies enrolled children with medically attended acute respiratory illness; however, the CDC study also enrolled adults, whereas our study was limited to children. The US Flu VE Network study required cough for enrollment and illness onset ≤7 days before enrollment; our study required evidence of fever and illness onset <5 days before enrollment. Although cough was not a required symptom in our study, it was reported in 84% of the children enrolled. Because the symptoms of children enrolled in the study were similar, it is unlikely that the differences in the VE point estimates can be explained by variations in study methods alone. Factors contributing to differences observed could include variation in study population and circulation of influenza strains, prior influenza exposure and vaccine history, prevalence of cross-reactive antibodies to drifted circulating influenza strains, and different IIV formulations and their match to circulating strains each year.

We are also aware of 2 studies that assessed the effectiveness of LAIV against influenza-associated hospitalizations among children in England and Scotland [31, 32]. LAIV was offered to all children as part of a national vaccination program. LAIV VE in children aged 2–6 years in England was 54.5% (95% CI, 31.5%–68.4%) for all influenza types combined, 48.3% (95% CI, 16.9%–67.8%) for influenza A(H1N1)pdm09, and 70.6% (33.2%–87.1%) for influenza B strains. Provisional results in children aged 4–11 years in Scotland showed that LAIV VE against any laboratory-confirmed cases of influenza was 63% (95% CI, 50%–72%).

As expected, children who received LAIV in our study were less likely to have high-risk medical conditions than those who received IIV. A sensitivity analysis that excluded high-risk children found that LAIV and IIV were effective against any influenza strains and against influenza A(H1N1)pdm09 strains. We controlled for other confounders, such as age group, number of outpatient visits in the last 6 months, health insurance status, and sex, in addition to site and date of enrollment.

Several factors may have contributed to lower LAIV VE in the 2015–2016 season. One potential explanation is the emergence of the influenza A(H1N1)pdm09 subclade 6B.1, which is poorly inhibited by postvaccination adult serum pools despite being antigenically similar based on ferret sera. These results suggest that antigenic evolution is occurring that is not detected by standard hemagglutinin inhibition assays [33]. Consistent with US surveillance data [16], all sequenced influenza A(H1N1)pdm09 viruses in our study belonged to subclade 6B.1 and were antigenically similar to the vaccine strain. Heat stability of the influenza A(H1N1)pdm09 vaccine strain has also been explored as a possible cause of lower LAIV VE. An investigation by the manufacturer of LAIV found that the A/California/7/2009 (H1N1)pdm09 strain used in the 2013–2014 season had an increased susceptibility to thermal degradation [24]. The A/Bolivia/559/2013 (H1N1)pdm09 strain used in the 2015–2016 season LAIV was more heat stable than previous season strains; however, VE was still lower than expected. The finding of lower VE led to the ACIP recommendation against using LAIV in the United States in the 2016–2017 season. LAIV is still recommended for use in Europe and Canada.

In conclusion, both LAIV and IIV provided statistically significant protection against any influenza among children aged 2–17 years in a geographically diverse US population in 2015–2016. Our findings also add to the clinical evidence suggesting that the effectiveness of LAIV against influenza A(H1N1)pdm09 strains has been lower than observed with IIV since the 2009 influenza A(H1N1) pandemic.

## Supplementary Data

Supplementary materials are available at *Clinical Infectious Diseases* online. Consisting of data provided by the authors to benefit the reader, the posted materials are not copyedited and are the sole responsibility of the authors, so questions or comments should be addressed to the corresponding author.

## Supplementary Material

Supplementary Table

## Notes

***Acknowledgments*.** We thank all the children and their caregivers who assented/consented to participate in this study and all the healthcare providers involved in the study. We thank Dayna Wyatt, RN, and Stephanie Longmire of Vanderbilt University Medical Center; Wanda Fikes, RN, CCRP, and Kempapura Murthy, MPH, of Baylor Scott & White Research Institute; and Lauren Vannoy of Wake Forest School of Medicine for their support with participant enrollment and data collection. We also thank David E. Tabor, PhD, Zhongying Chen, PhD, and Bin Lu, PhD, of MedImmune for evaluating the virus sequences. We thank inScience Communications, Springer Healthcare (New York, NY), for their technical expertise in the preparation of this manuscript, which was in accordance with Good Publication Practice (GPP3) guidelines and funded by MedImmune.

***Financial support*.** This study was supported by MedImmune.

***Potential conflicts of interest*.** K. A. P., T. R. P., E. A. B., M. R. G., S. A. I., P. K. K., H. Q. M., and K. R. have received research funding from MedImmune. H. C. and C. S. A. are full-time employees of MedImmune, a member of the AstraZeneca Group. B. C. is on the speakers’ bureau for Pfizer and Sanofi Pasteur and has received research funding from MedImmune. M. G. has received an institutional grant from the CDC for a related US Flu VE Network study and has received research funding from MedImmune. A. L. N. has received research funding from MedImmune and from Merck and Pfizer for unrelated studies. H. K. T. serves as an advisor for Sequirus and Vaxinnate and has received research funding from Gilead, MedImmune, and Sanofi Pasteur. All authors have submitted the ICMJE Form for Disclosure of Potential Conflicts of Interest. Conflicts that the editors consider relevant to the content of the manuscript have been disclosed.
