# Supplementary material for: 2015–2016 Vaccine Effectiveness of Live Attenuated and Inactivated Influenza Vaccines in Children in the United States
Source: Clin Infect Dis. 2017 Oct 4;66(5):665–72. doi: 10.1093/cid/cix869 (PMC5850007; doi:10.1093/cid/cix869)
Supplement: Supplementary Table [file cix869_suppl_supplementary_table.doc]

**Supplementary Table. Vaccine Effectiveness Estimates of LAIV, IIV3, and IIV4**

|  | **Distribution of Cases and Controls** | | | | | | | | | | **Vaccine Effectiveness Estimates  % (95% CI)** | | |
| --- | --- | --- | --- | --- | --- | --- | --- | --- | --- | --- | --- | --- | --- |
|  | **Unvaccinated Children (n = 594)** | | **LAIV Recipients (n = 101)** | | **IIV3 Recipients (n = 100)** | | **IIV4 Recipients (n = 202)** | | **IIV Missing Valence (n = 15)** | | **LAIV** | **IIV3** | **IIV4** |
|  | **Cases** | **Controls** | **Cases** | **Controls** | **Cases** | **Controls** | **Cases** | **Controls** | **Cases** | **Controls** |  |  |  |
| Any influenza | 173 | 421 | 22 | 79 | 11 | 89 | 30 | 172 | 3 | 12 | 46 (7–69) | 77 (52–89) | 59 (35–75) |
| A(H1N1)pdm09 | 102 |  | 12 |  | 9 |  | 16 |  | 0 |  | 50 (–2 to 75) | 81 (57–91) | 56 (18–76) |
| A(H3N2) | 1 |  | 1 |  | 0 |  | 0 |  | 0 |  | — | — | — |
| Any B strain | 70 |  | 9 |  | 2 |  | 14 |  | 3 |  | 47 (–18 to 76) | 65 (–67 to 93) | 60 (23–80) |
| Victoria | 48 |  | 3 |  | 1 |  | 7 |  | 1 |  | 69 (–7 to 91) | –16 (–1055 to 88) | 70 (29–87) |
| Yamagata | 15 |  | 6 |  | 1 |  | 6 |  | 1 |  | –9 (–223 to 63) | 76 (–112 to 97) | 29 (–119 to 77) |
| Missing lineage | 7 |  | 0 |  | 0 |  | 1 |  | 1 |  | *—* | — | — |

Abbreviations: CI, confidence interval; IIV3, trivalent inactivated influenza vaccine; IIV4, quadrivalent inactivated influenza vaccine; LAIV, live attenuated influenza vaccine.
